# Modeling One-Electron Oxidation Potentials and Hole Delocalization in Double-Stranded DNA by Multilayer and Dynamic Approaches

**DOI:** 10.1021/acs.jcim.4c00528

**Published:** 2024-06-10

**Authors:** Jesús Lucia-Tamudo, Sergio Díaz-Tendero, Juan J. Nogueira

**Affiliations:** † Department of Chemistry, Universidad Autónoma de Madrid, 28049 Madrid, Spain; ‡ Institute for Advanced Research in Chemistry (IAdChem), Universidad Autónoma de Madrid, 28049 Madrid, Spain; § Condensed Matter Physics Center (IFIMAC), Universidad Autónoma de Madrid, 28049 Madrid, Spain

## Abstract

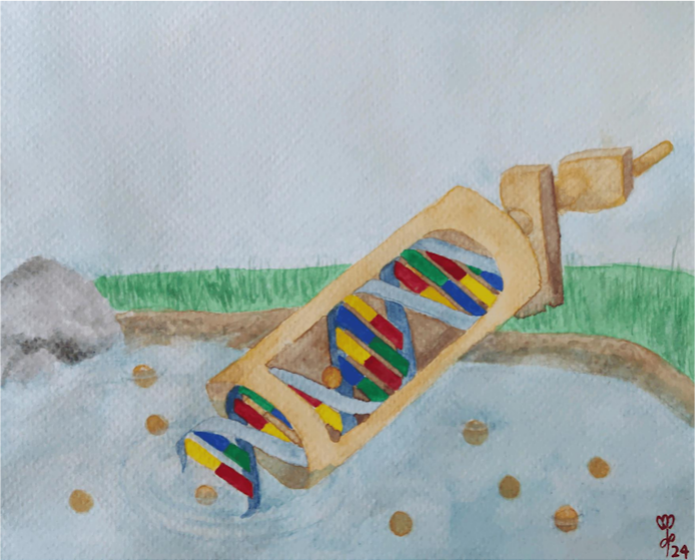

The number of innovative applications for DNA nowadays is growing quickly. Its use as a nanowire or electrochemical biosensor leads to the need for a deep understanding of the charge-transfer process along the strand, as well as its redox properties. These features are computationally simulated and analyzed in detail throughout this work by combining molecular dynamics, multilayer schemes, and the Marcus theory. One-electron oxidation potential and hole delocalization have been analyzed for six DNA double strands that cover all possible binary combinations of nucleotides. The results have revealed that the one-electron oxidation potential decreases with respect to the single-stranded DNA, giving evidence that the greater rigidity of a double helix induces an increase in the capacity of storing the positive charge generated upon oxidation. In addition, the hole is mainly stored in nucleobases with large reducer character, i.e., purines, especially when those are arranged in a stacked configuration in the same strand. From the computational point of view, the sampling needed to describe biological systems implies a significant computational cost. Here, we show that a small number of representative conformations generated by clustering analysis provides accurate results when compared with those obtained from sampling, reducing considerably the computational cost.

## 1 Introduction

The applications of DNA have evolved from storing genetic information in living organisms to having innovative applications such as DNA computation, DNA-templated synthesis, molecular detection, and the use of DNA as a nanowire.^1^ DNA computation uses DNA as a molecular computer by leveraging the hybridization ability of DNA strands.^2,3^ DNA-templated synthesis utilizes DNA as a template to synthesize materials with specific properties.^4^ Molecular detection allows for the detection of specific molecules through DNA hybridization or redox reactions between the analyte and a nucleeobase.^5−15^ In addition, using DNA as a nanowire enables the construction of electronic devices based on DNA strands.^16,17^

Regarding molecular detection and DNA nanowires, charge transfer between the DNA strand and analyte/electrode is a crucial process. Nucleobases play a primary role in charge transfer, and obtaining precise values of redox properties like one-electron oxidation potential is essential since they usually undergo oxidation rather than reduction.^18−31^ The reducer ability of nucleobases in water follows a specific order: G > A > T ∼ C > U^32^ (Purines: G = guanine and A = adenine; pyrimidines: T = thymine, C = cytosine, and U = uracil). In fact, we have shown a clear correlation between the number of atoms in a nucleobase that participate in delocalizing the positive charge and the relative reducer character of nucleobases in a previous work.^31^ After nucleobase oxidation, hole transport along the DNA strand occurs, and nucleobases contribute to translate the positive charge. Two proposed mechanisms, tunneling and hopping, explain this charge transport.^33−36^ Tunneling involves hole delocalization along multiple nucleobases, while hopping is a multistep process where the charge is localized in one nucleobase and moves through consecutive jumps. From previous works, it has been suggested that hopping is the predominant mechanism for single-stranded DNA (ss-DNA) in the electronic ground state, mainly due to solvent effects that stabilize charge delocalization.^32,37−39^ Additionally, also in ss-DNA, the charge tends to be held in a single nucleobase or, in some cases, in more than one, depending on the solvent and nucleobase type. Some of our previous works have focused on studying the redox properties and hole distribution in ss-DNA molecules. However, these aspects have not been discussed for double helices yet, although previous works have been carried out to determine the electronic structure and the mobility of an electron or a hole in these structures.^40,41^

The most important disadvantage of modeling biological systems with a large number of degrees of freedom is the high computational cost that is required. Since there is a large number of geometries to be considered from the sampling, the calculations involve a significant amount of time. In order to overcome this limitation, there exist some techniques that reduce the number of geometries to analyze, e.g., clustering analysis. This methodology classifies the total number of conformations in a specific number of groups in terms of the similarity between geometries, although they can also be classified based on the similarity in a specific property. This means that different snapshots from the dynamic simulations with similar geometry form a collective of frames, called cluster, which are considered to have similar properties. This clustering classification is often performed based on the calculation of the root-mean-square deviation (rmsd) between all configurations. Then, the centroid structure of each cluster can be determined, and, as an approximation, one can consider that the properties of the centroid structure will accurately represent the properties of the whole ensemble of geometries composing the cluster. As a result, the number of conformations to be taken into account in the calculations of the desired property is considerably reduced.

**Figure 1:**
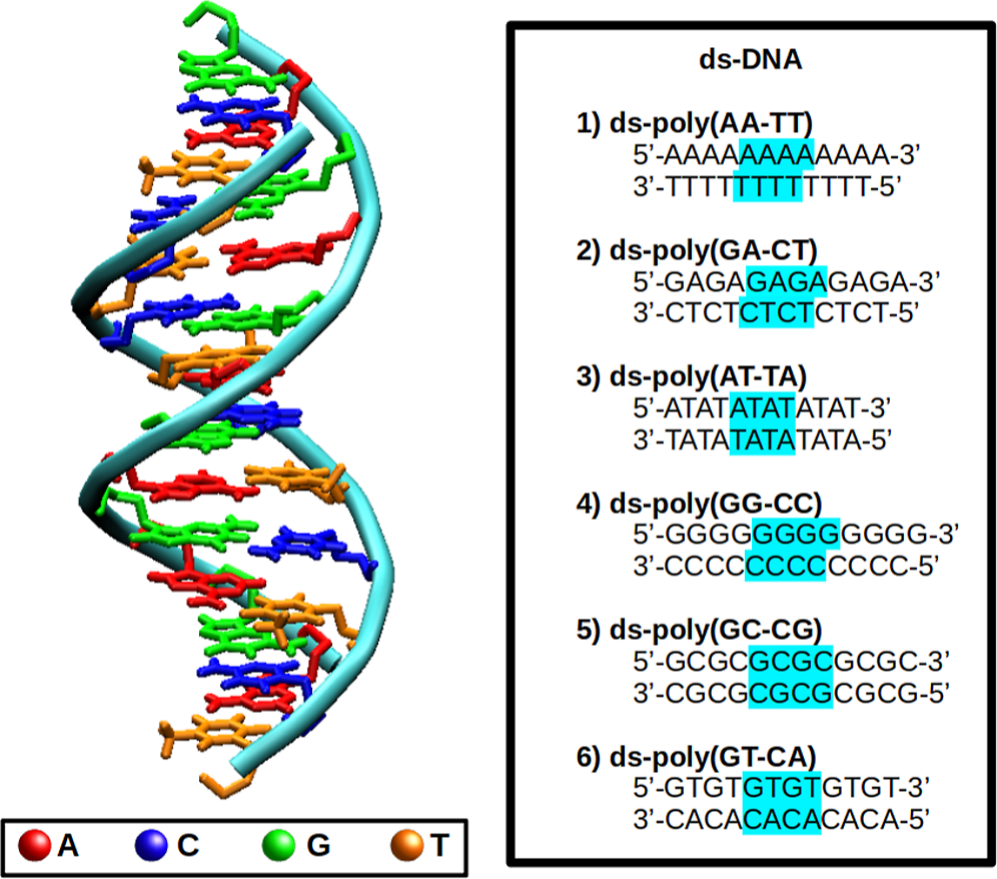
Graphical view of the general form of the systems under study. Each nucleobase is associated with a color. The full sequence for each system is displayed in the right panel. Cyan refers to the nucleobases that form the CAM-B3LYP layer and to the sugar and phosphate described in the xTB layer.

In this work, we have determined the one-electron oxidation potential and the hole delocalization along the strand of all possible binary combinations of model double-stranded DNA (ds-DNA) (see Figure 1). In addition, we discuss the different factors that influence the magnitude of these two properties. Finally, a clustering study has been conducted in order to determine whether the redox properties computed for a reduced number of representative conformations are in good agreement with the properties computed for a large ensemble of geometries.

## 2 Methods and Computational Details

The computation of the one-electron oxidation potential and the delocalization properties of the strands were conducted using a similar procedure to the one employed in previous studies on ss-DNA.^32,39^ A conformational sampling was carried out using classical molecular dynamics (MD) followed by quantum-mechanics/molecular-mechanics (QM/MM) MD simulations. When dealing with large-size systems, the introduction of conformational sampling is needed to populate all relevant potential energy minima. The choice of an arbitrary minimum from an optimized structure could lead to wrong results as previously shown when analyzing the excited electron delocalization in DNA strands.^42^ After conformational sampling, the properties were computed from a selected ensemble of geometries, obtained from the sampling, through electronic-structure calculations. These calculations were performed using a QM1/QM2/Continuum approach in combination with the Marcus theory. In the following, the computational details of this protocol are explained.

The nab application provided by the AmberTools 22 package^43−45^ was used to build the initial geometries of the ds-DNA strands. Each double helix was composed of 24 nucleotides arranged in a specific pattern, as shown in Figure 1. Thus, each strand was composed of 12 nucleotides. In order to analyze all possible binary combinations of nucleotides in one strand, six ds-DNA models were built where the complementary strand fully matched with the one with direction 5′ → 3′ (see Figure 1). The ds-DNA molecules were solvated in a truncated octahedron box with a buffer of 16 Å, and the tleap program implemented in AmberTools 22 was used for this purpose. The ff90bsc0 force field^46,47^ in combination with the bsc1 dihedral correction^48^ was employed to describe the DNA molecules, while the TIP3P force field^49^ modeled the interaction description of the water molecules. To counteract the negative charge of each strand, 22 sodium cations were added using the parameters described by Joung and Cheatham.^50^

The exploration of the configurational space was conducted through classical MD simulations^51−53^ using the CUDA version of the pmemd program, which is implemented in the AMBER 20 package.^43−45^ The simulations began with a 10000-step minimization, where the steepest-descent algorithm was used for the first 5000 steps,^54^ followed by the Newton–Raphson algorithm for the subsequent 5000 steps.^55^ To regulate the temperature, a constant volume (*NVT*) progressive heating up to 300 K was performed for 500 ps, applying a Langevin thermostat with a collision frequency of 2 ps^–1^. Afterward, an additional 500 ps simulation was conducted at a constant temperature of 300 K (using a canonical (*NVT*) ensemble). In the next phase, a 1 ns simulation was run in the isothermal–isobaric (*NPT*) ensemble to balance the system volume and achieve the desired density. Finally, a production simulation of 200 ns was performed in the (*NPT*) ensemble, and 200 equidistant snapshots were fetched. Throughout all simulations within the (*NPT*) ensemble, the Berendsen barostat with isotropic position scaling and a pressure relaxation time of 2 ps was employed to maintain a constant pressure of 1 bar. During the entire dynamic protocol, the particle-mesh Ewald method with a grid spacing of 1.0 Å was used to compute electrostatic interactions, while a cutoff of 10 Å was applied for nonbonded interactions. The SHAKE algorithm^56−58^ was utilized to constrain the hydrogen-containing bonds, and a time step of 2 fs was used for the heating, equilibration, and production stages.

From the classical MD simulations, 200 geometries were selected for each strand as initial conditions to run QM/MM MD simulations in order to refine the structure of the relevant region of the system. These simulations were carried out for both the neutral and the cationic strands to apply in the next step, the Marcus model. These QM/MM MD simulations were evolved for 300 steps in the (*NPT*) ensemble using the ORCA/AMBER interface.^59^ The QM region comprises eight nucleobases, four adjacent nucleobases in one strand and their complementary ones in the opposite, and was described using the xTB model^60^ and the 6-311G(d)^61,62^ basis set. The use of a QM/MM protocol to describe the strand in the dynamics is more accurate than the use of a force field, especially for the cationic strand, for which the force field should ideally be parametrized. Finally, the last geometry obtained from each QM/MM MD simulation was employed to compute the vertical ionization energy (VIE) and the vertical attachment energy (VAE) for each system. Using the Marcus theory,^63−68^ the one-electron oxidation potential  was computed using eq 1
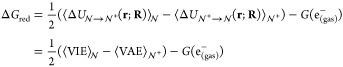
1to calculate the Gibbs free energy from its average VIE and VAE. Notice that *G*(e_(gas)_^–^) is the free energy of the electron in the gas phase. In addition, this free energy can be related to the redox potential through eq 2.
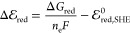
2where  refers to the reduction potential of the standard hydrogen electrode, the reference electrode selected for this work.^32,39^ In fact, the term *G*(e_(gas)_^–^) in eq 2 is included because it is also taken into account in the value of  V, which is extensively employed in the literature.^31,69−72^

These calculations were performed employing a hybrid QM1/QM2/Continuum approach, where the QM1/QM2 interaction was described by an electrostatic embedding. Specifically, the VIEs and VAEs were determined for the QM1 region, consisting of the eight nucleobases previously mentioned, using the CAM-B3LYP/6-311G(d) level of theory, similarly to other previous works.^31,32,39^ As show in Figure S1 of the Supporting Information, the inclusion of eight nucleobases in the QM1 is enough to get converged results. The nucleotides with nucleobases that are not involved in the QM region were excluded from the final calculation, while the phosphates and sugars of the QM nucleobases were in the second layer QM2 described by the DFTB approach with the GFN2-xTB scheme.^60^ The effects of the solvent were accounted for using the ALPB continuum solvation model,^73^ which is compatible with DFTB. All computations were carried out using the ORCA 5.0.3 package.^59^

To analyze the localization of the hole, the molecular charge difference of each nucleobase in the QM1 region upon ionization of the neutral species in each geometry was calculated. Löwdin charges^74^ were employed for charge calculations, and analysis was conducted using custom scripts. The intermolecular delocalization number, denoted as *n*, was defined as the number of nucleobases among which the positive charge is distributed after ionization. To determine *n*, the eight nucleobases considered in the QM1 region were first ordered based on increasing hole charge, and then an empirical eq (eq 3) was applied. The technical explanation and the details of this empirical equation can be found in ref (32).
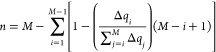
3

In a similar spirit, Pipek and Mezey proposed an alternative method to quantify the delocalization of a positive charge within a system.^75^ They derived an index using the gross atomic Mulliken population of the set of orbitals in each atom. In order to compare our empirical eq 3, we adapted their formula by incorporating the partial charge of each nucleobase in the considered QM region (see eq 4).
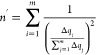
4

Finally, a clustering analysis was carried out to investigate whether reducing the number of geometries could yield similar results to studying the complete set of geometries considered throughout the trajectories. This analysis was performed using the cpptraj tool implemented in the AMBER 20 package.^43−45^ Thus, a convergence analysis was conducted to determine the number of clusters required to obtain a converged value of VIE, VAE, , and *n* (in both neutral and cationic trajectories).

## 3 Results

### 3.1 One-Electron Oxidation Potential

We begin the discussion by examining the redox power of the systems addressed in this work. To this end, the one-electron oxidation potentials of the double helices have been determined using the above-described computational protocol. In a previous work, such potential was studied in homogeneous ss-polyX systems, where the following reducing capacity order was concluded: ss-polyG > ss-polyA > ss-polyT > ss-polyC.^32^ This relative order was related to the extent of the π-system of each nucleobase. In this way, those strands derived from purine nucleobases (G and A), with a larger π-system, exhibited greater reducing power, i.e., a lower one-electron oxidation potential than the strands formed by pyrimidine bases. Additionally, we also studied heterogeneous ss-polyXY systems with binary combinations of nucleotides.^39^ The simulations showed that the resulting one-electron oxidation potential was, roughly speaking, a linear combination of the potentials of the homogeneous strands.

**Figure 2:**
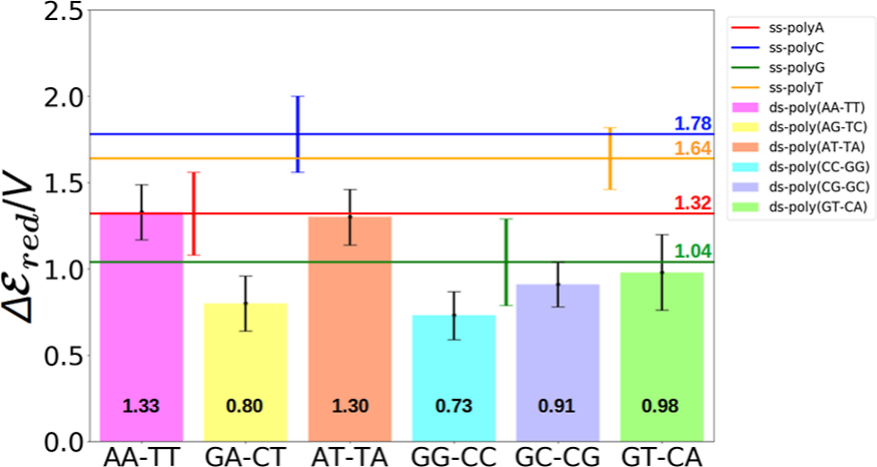
One-electron oxidation potential predicted for ss-polyX (solid lines) and ds-poly(XY-X′Y′) (bars) in aqueous phase. Black values inside the bars are the one-electron oxidation potential of the corresponding ds-DNA models, while colored values next to the horizontal lines are those for homogeneous ss-DNA. Standard deviations for each strand is represented by vertical lines. Notice that  for ss-polyX are taken from ref (32).

We will refer to the ds-DNA molecules investigated in this work as ds-poly(XY-X′Y′), where X and Y represent the binary combination of nucleobases appearing in the 5′ → 3′ direction strand. The complementary strand 3′ → 5′ is thereby determined since the systems have been modeled without mismatches. Thus, X′ and Y′ are the complementary nucleobases of X and Y, respectively. In the case of ds-poly(XY-X′Y′) systems, the reducing power is presented in Figure 2 in terms of the one-electron oxidation potential. Generally, there is a noticeable decrease in the oxidation potentials compared to the ss-polyXY systems studied previously.^32,39^ Moreover, the trend observed in the case of heterogeneous ss-DNA, where the redox potential was a linear combination of the potentials of the pure strands weighted by the abundance of each nucleobase in the heterogeneous strand, is not observed. Therefore, the behavior exhibited by ss-DNA systems cannot be extended to ds-DNA systems. This indicates that the intermolecular interactions between strands lead to a greater stabilization of the resulting positive charge, significantly increasing its capacity to undergo oxidation. This larger stabilization of the generated positive hole can be attributed to the larger rigidity of ds-DNA with respect to ss-DNA, which would allow delocalization in consecutive stacked nucleobases (π – π stacking). On the contrary, it may be due to the possibility of delocalizing the charge in two nucleobases, paired through hydrogen bonds (G-C; A-T), and establishing a large π region to hold it. These assumptions are further investigated in the next section, where the charge delocalization along the DNA strand is analyzed.

There are certain patterns common to all single strands. A careful analysis of Figure 2 reveals a difference in the potential between strands with and without guanine bases. The one-electron oxidation potential is significantly higher in DNA molecules that lack guanine nucleobases. This is consistent with the hierarchy of reducing power among nucleobases, where guanine tops the list. Therefore, it can be stated that guanine plays a predominant role in increasing the reducing power of a ds-poly(XY-X′Y′) system, in the same way it does in ss-DNA. If we compare the two systems in which guanine is absent [ds-poly(AA-TT) and ds-poly(AT-TA)], we can observe that the redox potential is practically identical. Therefore, there does not appear to be a relationship between the sequence of the strands and the potential, but rather between the overall composition (abundance of each nitrogenous base) and the potential. On the other hand, the situation is clearly different when guanine is present. First, ds-poly(GG-CC) and ds-poly(GC-CG) have the same percentage of guanines, but in the first one the guanines occupy adjacent positions in the same strand. This distribution seems to favor the oxidation process of the strand since the oxidation potential of ds-poly(GG-CC) is smaller than that of ds-poly(GC-CG), where the guanines are arranged diagonally in opposite strands (0.73 vs 0.91 V). The same trend is observed for ds-poly(GA-CT) and ds-poly(GT-CA) if one focuses on the position of the purine nucleobases. In ds-poly(GA-CT), the purines guanine and adenine are adjacent to each other in the same strand and, consequently, it has a greater reducing power (lower oxidation potential) than ds-poly(GT-CA) (0.80 vs 0.98 V). Therefore, it seems that the position of the nucleobases in the strands does have a relevant effect when guanine is present, while it is not important in other cases.

### 3.2 Charge Delocalization along the DNA Strand

In order to understand the differences in one-electron oxidation potentials based on the arrangement of nucleobases along a strand, we studied the distribution of holes among the nucleobases considered in the QM region. As explained earlier, oxidation results in the generation of a positive charge in the DNA double helix. This charge can be delocalized along the strand among several nucleobases or localized in only one of them. Precisely, the two most accepted mechanisms for charge transport in DNA molecules revolve around this idea. On one hand, tunneling advocates for transport based on the delocalization of the positive charge among several nucleobases simultaneously, evolving over time from one side of the strand to the other. On the other hand, the hopping mechanism states that transport occurs through jumps of the localized hole from one nucleobase to another. Therefore, studying the distribution of the hole among the nucleobases of the considered systems could help elucidate the dominant mechanism in DNA charge transport.

**Figure 3:**
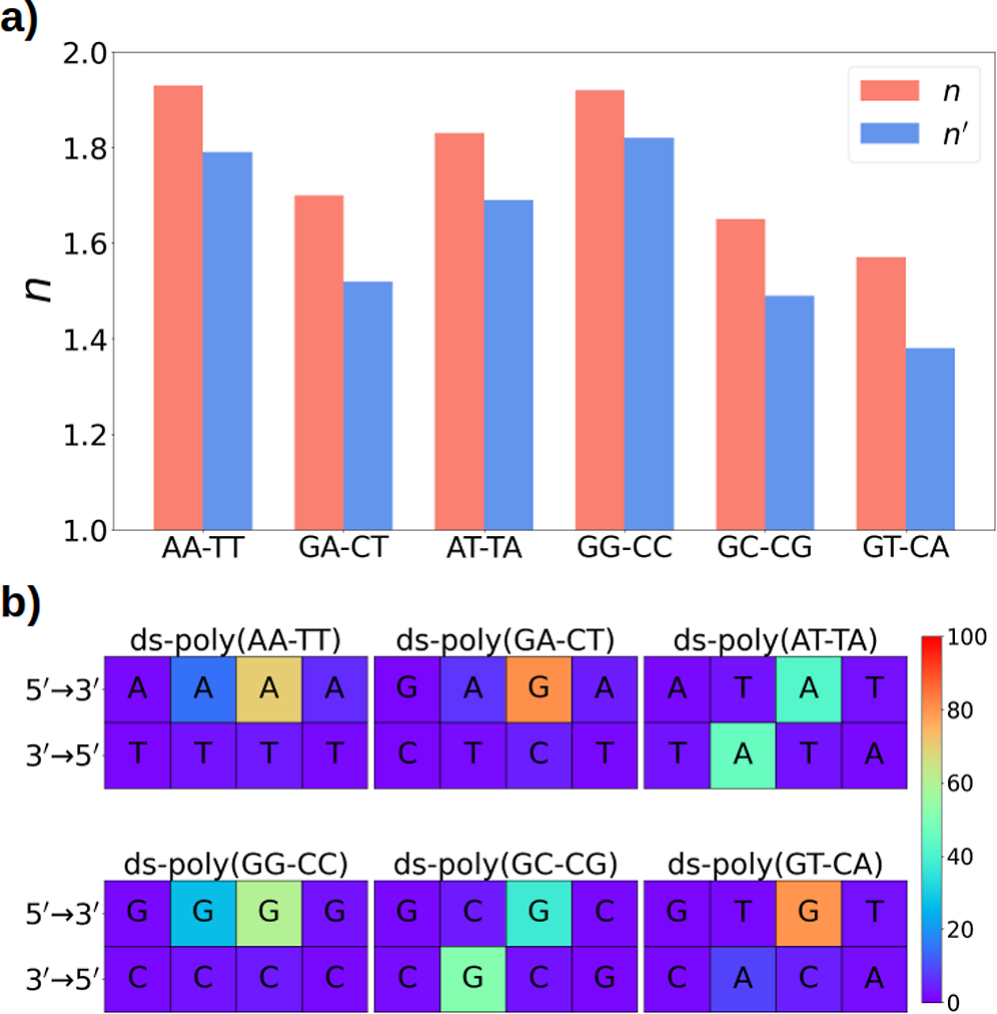
(a) Intermolecular delocalization number *n* (red) or *n*′ (blue) for each ds-DNA system considered. (b) Graphical representation of the percentage of the amount of positive hole stored in each nucleobase of a specific system.

Figure 3 shows the charge distribution analysis along each double helix investigated here. Specifically, Figure 3a displays the two different delocalization numbers explained above, *n*′ and *n*. It can be seen that, although the value of *n*′ extracted from each system (see eq 4) is lower than that of *n* (see eq 3), the relative delocalization order among strands is maintained independently on the way of computing the delocalization number. In general, the variation of the intermolecular delocalization numbers is less important for double strands than for single strands. All *n* values for ds-DNA fall within the range of 1.6–2.0, while in the case of ss-DNA it has been previously computed a slightly wider interval range going from 1.56 to 2.40.^39^ The explanation for this behavior is related to the composition of the double helix. In ds-DNA, the number of purines (large delocalization) always equals the number of pyrimidines (small delocalization) and, therefore, the obtained *n* values are not as large as those reached in ss-DNA, where only purines can be present.

Figure 3a,b shows that the positive charge is mainly located on the most reducing nucleobase, i.e., guanine, or on adenine when guanine is not part of the strand. In addition, when two purines are stacked in the same strand the charge delocalization is larger than when they are located in a diagonal arrangement in different strands. This can be observed when comparing *n* for ds-poly(AA-TT) vs ds-poly(AT-TA) (1.93 vs 1.83), ds-poly(GG-CC) vs ds-poly(GC-CG) (1.92 vs 1.65), and ds-poly(GA-CT) vs ds-poly(GT-CA) (1.70 vs 1.57). This can be easily explained by the more efficient orbital overlap between adjacent stacked nucleobases than between diagonal interacting ones, allowing for a larger delocalization of the positive hole.

A combined analysis of Figures 2 and 3 reveals that there is no direct correlation between the one-electron oxidation potential and the intermolecular delocalization number, as could be expected. Instead, as we previously showed,^32^ the reducing power of the strands is dominated by the intramolecular hole delocalization within a single nucleobase rather than the intermolecular one. In other words, the magnitude of the oxidation potential is dominated by the specific nature of the nucleobases composing the strand. For example, the double strands with the lowest oxidation potentials are those that contain guanine, while the guanine-free strands, ds-poly(AA-TT) and ds-poly(AT-TA), present larger potentials (see Figure 2). The intermolecular delocalization is more related to the reduction of the oxidation potential when going from the isolated nucleobase to the strand.^32^ Therefore, a correlation between the oxidation potential and the intermolecular delocalization number *n* is found only when strands having the same nucleobase composition are compared. Specifically, the lower oxidation potentials (larger reducing power) are related to slightly larger hole intermolecular delocalization numbers, as shown by, for example, ds-poly(GA-CT) ( = 0.7 V and *n* = 1.7) vs ds-poly(GT-CA) ( = 0.9 V and *n* = 1.6) or ds-poly(GG-CC) ( = 0.7 V and *n* = 1.9) vs ds-poly(GC-CG) ( = 0.9 V and *n* = 1.7).

In all ds-DNA strands investigated, it is observed that the positive charge storage occurs mainly in purines, and delocalization does not extend to more than two nucleobases. These features are typical of the mechanism known as hopping, in which transport occurs through jumps between nucleobases. However, since the hole charge is not completely located on one nucleobase, the tunneling mechanism cannot be completely ruled out and it is very likely that both mechanisms might operate simultaneously.

### 3.3 Clustering Analysis

**Figure 4:**
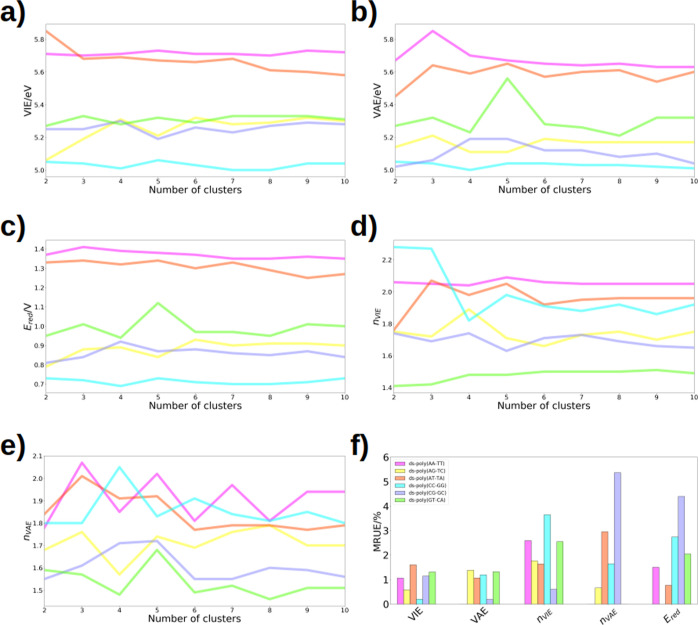
Convergence of (a) VIE, (b) VAE, (c) , (d) *n*_VIE_, and (e) *n*_VAE_ with respect to the number of clusters. (f) Bar plot of the MRUEs for each system in terms of the VIE, VAE, delocalization number for the neutral and cationic species (*n*_VIE_ and *n*_VAE_), and one-electron oxidation potential. The MRUEs are calculated with respect to the values obtained from the dynamics using 200 snapshots.

The results discussed in previous sections require the computation of redox properties on a large number of geometries for each system, leading to a significantly high computational cost. To investigate the possible reduction of such cost, we performed a clustering analysis. This technique classifies the ensemble of configurations in terms of the rmsd between their geometries. Conformations separated in the configuration space by small (large) rmsd will belong to the same (different) cluster. Finally, the structure corresponding to the centroid of each cluster is computed and associated with the closest configuration of the system, which becomes the representative conformation of the cluster. The properties of the cluster, therefore, are associated with the properties of the representative geometry. Thus, the total set of geometries from the dynamics for each system was grouped into a certain number of clusters, varying from 2 to 10, and the properties were calculated only for the representative structures of these clusters (see Figure 4a–e). Specifically, for the trajectories of the neutral (cationic) DNA strands, the VIE (VAE) and the delocalization number *n*_VIE_ (*n*_VAE_) were computed. Then, the average value of each property  is determined using eq 5, where the values of the property in each centroid  of each cluster *i* = 1, ..., *l* are weighted by the fraction of geometries included in each cluster (*P*(*i*))
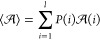
5

Figure 4 shows the variation of each computed property as a function of the number of representative structures considered in the calculation. In the case of ds-poly(AA-TT) and ds-poly(GA-CT), convergence is reached after considering 7 geometries in the calculation, while for the remaining systems (in both neutral and cationic trajectories), 6 representative geometries are sufficient. Once convergence was achieved, the average values of VIE, VAE, and the intermolecular delocalization number of the neutral (*n*_VIE_) and cationic (*n*_VAE_) systems were determined. The resulting values of the VIE and the VAE were used to calculate . Figure 4f shows the mean relative unsigned error (MRUE) of these properties with respect to those obtained from the complete trajectory, employing 200 snapshots, as explained above. It can be observed that these errors are considerably low, not exceeding 6%. This reflects that the value of all these properties can be estimated quite accurately using only a small number of representative geometries from the dynamics, specifically with 6–7 conformations. In conclusion, the redox properties of DNA strands can be studied with relatively low computational effort, allowing application of high-level electronic-structure methods to obtain more accurate properties.

## 4 Conclusions

An extensive computational study has been carried out on the redox properties of DNA double-helix model systems, as well as on how a vacancy is distributed along this structure. In general terms, a significant increase in the reducer character of the nucleobases has been observed when they are part of a DNA double strand, compared to when they are arranged in a single strand. Intermolecular interactions between bases from different strands are capable of stabilizing to a greater extent the hole generated in the oxidation process, thereby facilitating the occurrence of this phenomenon. Moreover, a clear relationship between the reducing power of the strands and their nucleobase composition and arrangement has been observed. Thus, helices with a higher percentage of purines are more reducing than those with a higher percentage of pyrimidines, especially when the purines are stacked in the same strand rather than in a diagonal disposition in different strands.

The intermolecular delocalization number has proven to be relatively small for ds-DNA (always smaller than 2). In fact, intermolecular delocalization is smaller than in the case of single strands, due to the presence of both purine and pyrimidine nucleobases. In general, the positive charge is localized exclusively on purine-based nucleobases, which is consistent with the presence of a more extended π-system in comparison with pyrimidines. The charge, therefore, is delocalized mainly between only two adjacent purine nucleobases (stacked in the same strand or diagonally arranged), making the hopping mechanism predominant, as outlined in previous works for ss-DNA. Furthermore, when the adjacent purine nucleobases are different, there is a clear predominance of guanine in hosting most of the positive charge. Moreover, for strands with the same nucleobase composition, larger intermolecular delocalization of the hole is related to smaller one-electron oxidation potentials, i.e., to a stronger reducing power.

Finally, it has been demonstrated that the number of geometries to be considered in this type of calculations can be reduced using a clustering approach. With a very small number of conformations (6 or 7), the redox properties are satisfactorily similar to those obtained with a larger number of frames (200) selected from the trajectories. Thus, higher level electronic-structure methods can be applied to these representative geometries in order to obtain more accurate results.

## Data Availability

The nab and tleap toolkits from the AmberTools 22 package^43−45^ were used to generate the topology and coordinate files for the MD simulations. The PMEMD.CUDA module of the AMBER 20^76,77^ (https://ambermd.org/) software was used to perform the classical MD simulations. Afterward, the SANDER module of the AMBER 20 software^43−45^ was used in combination with the ORCA 5.0.3 package^59^ (https://orcaforum.kofo.mpg.de/app.php/portal) to conduct the QM/MM MD simulations. With the aim of automatically generating the QM/MM input files, the MoBioTools toolkit (https://github.com/mobiochem/MoBioTools) was used.^78^ QM1/QM2/ALPB computations for the redox properties and hole delocalization were performed with the ORCA 5.0.3 software.^59^ Homemade scripts were generated with Python 3 (https://www.python.org/) in order to analyze the hole delocalization of the strands. The cpptraj application from the AmberTools 22 package was used to perform clustering analyses. Finally, trajectories were visualized with the Visual Molecular Dynamics (VMD, https://www.ks.uiuc.edu/Research/vmd/).^79^

## Supporting Information Available

Convergence of the QM1 region size, root mean squared displacement of the ds-poly(GC) strand along the classical dynamics, and time evolution of the inter pair-base distance for the ds-poly(GC) strand along the QM/MM dynamics (PDF)
