# Supplementary material for: Modeling One-Electron Oxidation Potentials and Hole Delocalization in Double-Stranded DNA by Multilayer and Dynamic Approaches
Source: J Chem Inf Model. 2024 Jun 10;64(12):4802–10. doi: 10.1021/acs.jcim.4c00528 (PMC11200263; doi:10.1021/acs.jcim.4c00528)
Supplement: Supplementary file 1 — ci4c00528_si_001.pdf [file ci4c00528_si_001.pdf]

Supporting Information of "Modelling One-Electron Oxidation Potentials and Hole Delocalization in Double-Stranded DNA by Multilayer and Dynamic Approaches"

Jesús Lucia-Tamudo,† Sergio Díaz-Tendero,*,†,‡,¶ and Juan J. Nogueira*,†,‡

†*Department of Chemistry, Universidad Autónoma de Madrid, 28049, Madrid, Spain*

‡*Institute for Advanced Research in Chemistry (IAdChem), Universidad Autónoma de Madrid, 28049 Madrid, Spain*

¶*Condensed Matter Physics Center (IFIMAC), Universidad Autónoma de Madrid, 28049 Madrid, Spain*

E-mail: sergio.diaztendero@uam.es; juan.nogueira@uam.es

1 Convergence of the Vertical Ionization Energy with the QM1 Region Size

In Figure S1, the value of the VIE for a snapshot taken from neutral trajectory is computed as function of the size of the QM1 region. The different sizes considered were from one to four pair bases for the ds-poly(GG-CC) model, which base pairs are identical along the double strand. As it can be observed, the VIE decreases with the number of base pairs included in the QM1 region since more positive charge delocalization is allowed upon ionization. However, when three base pairs are included, the value of the VIE is converged. This is in

good agreement with the charge delocalization analysis (see Figure 3 of the main manuscript), which predict an intermolecular delocalization number not higher than 2. Thus, a QM region including four nucleobases is enough to get converged results.

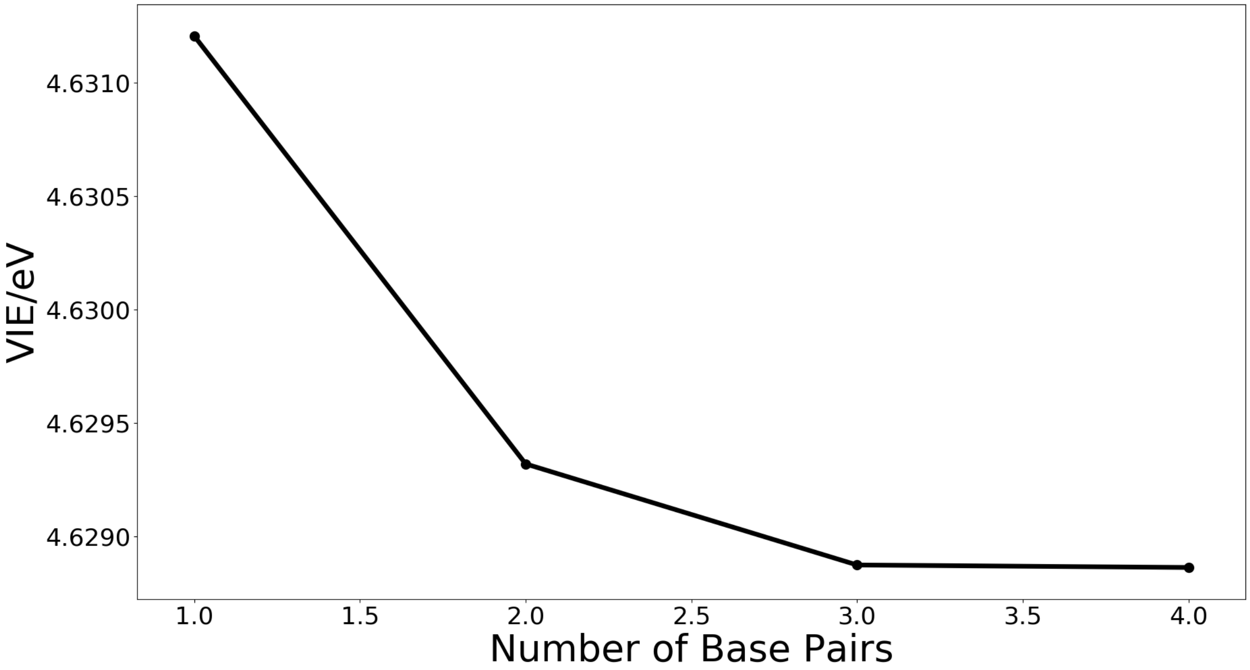

Figure S1: VIE values in terms of the number of base pairs included in the QM1 region. Results are obtained from one neutral snapshot from the ds-poly(GG-CC) model.

2 RMSD of the Classical Trajectory

The RMSD of the ds-poly(GG-CC) strand oscillated around a constant value, showing that the structure of the system is well equilibrated.

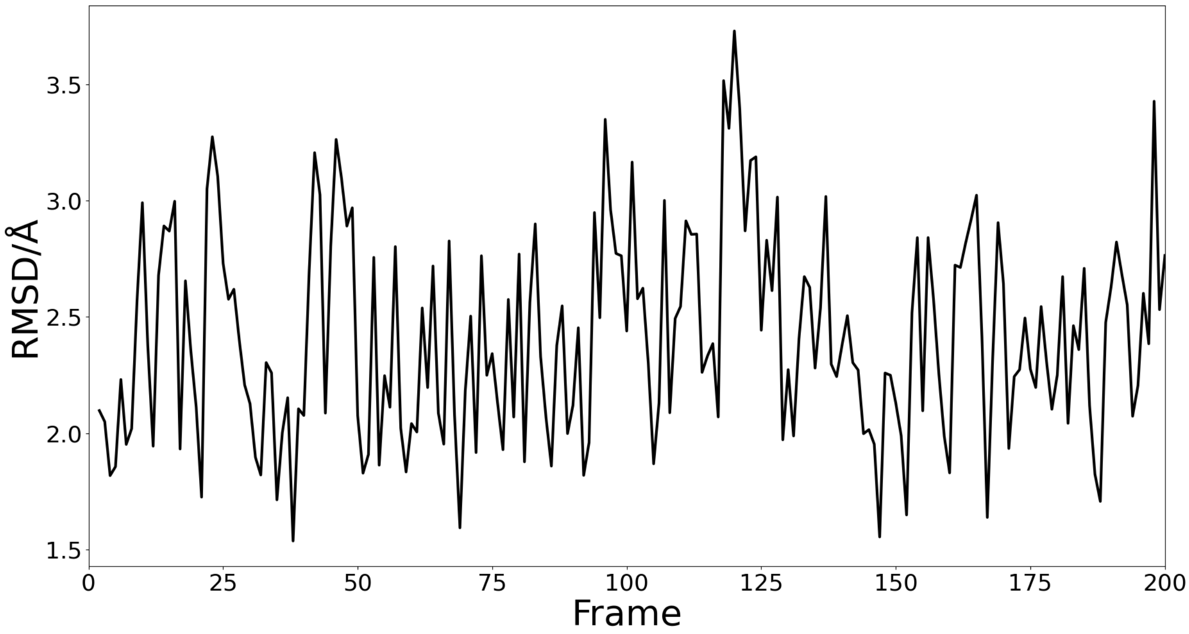

Figure S2: Root mean square deviation of the ds-poly(GG-CC) model along the classical dynamics simulation.

3 Inter Pair-Base Distance Distribution of the QM/MM MD Sampling

The time evolution of the interbase distance shows that the description of the nucleobases at QM level (colour lines) allows for a better sampling since the nucleobases are able to approach to each other closer than when the classical force field is employed (black line). This can be explained by the better description of polarization effects by the DFT functional in the QM/MM protocol than by the fixed-charged force field employed in the classical simulations.

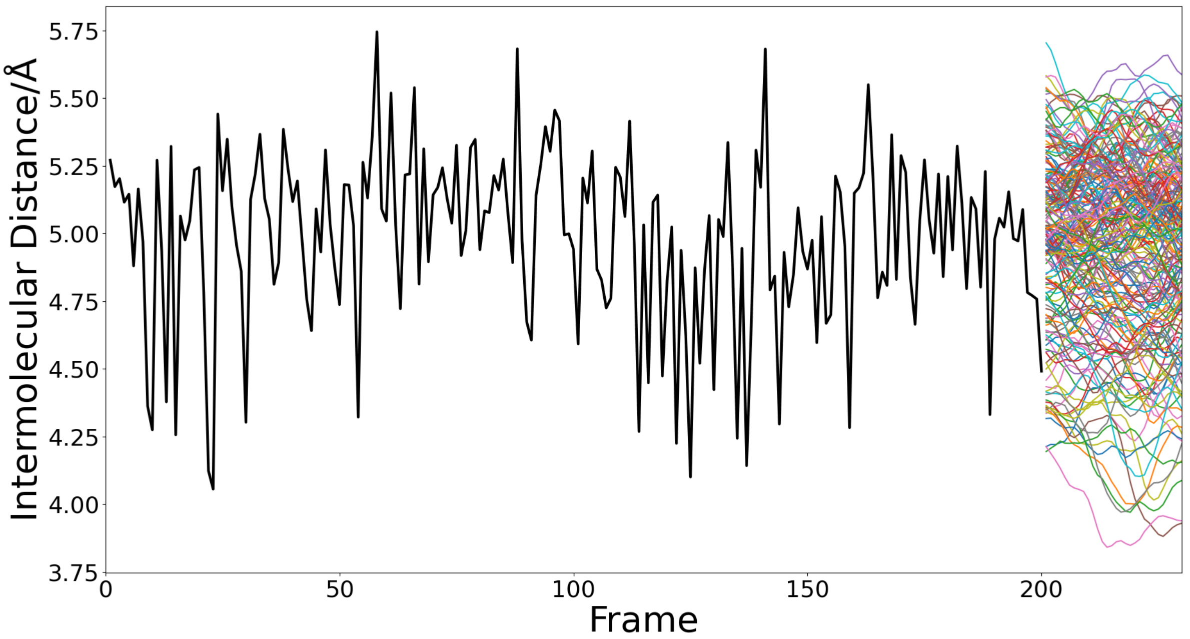

Figure S3: Conformational sampling of the inter-base pair distance in the ds-poly(GG-CC) model. Black line corresponds to the classical molecular dynamics simulation while coloured lines represent the neutral trajectories of the 300-step QM/MM MD simulations performed from classical geometries.
